# Leveraging the trust of nurses to advance a digital agenda in Europe: a critical review of health policy literature

**DOI:** 10.12688/openreseurope.13231.1

**Published:** 2021-03-26

**Authors:** Paul De Raeve, Patricia M. Davidson, Franklin A. Shaffer, Eric Pol, Amit Kumar Pandey, Elizabeth Adams

**Affiliations:** 1 European Federation of Nurses Associations, Brussels, 1050, Belgium; 2 Johns Hopkins School of Nursing, Baltimore, 21205, USA; 3 CGFNS International, Inc., Philadelphia, 19104-2651, USA; 4 aNewGovernance, Brussels, 1050, Belgium; 5 Socients AI and Robotics (SAS), 185 RUE DES GROS GRES, Colombes, 92700, France

**Keywords:** Electronic Health Record, Artificial intelligence, co-creation, co-design, nurses, nursing care, health, healthcare, policy.

## Abstract

This article is a critical and integrative review of health policy literature examining artificial intelligence (AI) and its implications for healthcare systems and the frontline nursing workforce. A key focus is on co-creation as essential for the deployment and adoption of AI. Our review hinges on the European Commission’s White Paper on Artificial Intelligence from 2020, which provides a useful roadmap. The value of health data spaces and electronic health records (EHRs) is considered; and the role of advanced nurse practitioners in harnessing the potential of AI tools in their practice is articulated. Finally, this paper examines “trust” as a precondition for the successful deployment and adoption of AI in Europe.

AI applications in healthcare can enhance safety and quality, and mitigate against common risks and challenges, once the necessary level of trust is achieved among all stakeholders. Such an approach can enable effective preventative care across healthcare settings, particularly community and primary care. However, the acceptance of AI tools in healthcare is dependent on the robustness, validity and reliability of data collected and donated from EHRs (Smart4Health & InteropEHRate). Nurse stakeholders have a key role to play in this regard, since trust can only be fostered through engaging frontline end-users in the co-design of EHRs and new AI tools. Nurses hold an intimate understanding of the direct benefits of such technology, such as releasing valuable nursing time for essential patient care, and empowering patients and their family members as recipients of nursing care.

This article brings together insights from a unique group of stakeholders to explore the interaction between AI, the co-creation of data spaces and EHRs, and the role of the frontline nursing workforce. We identify the pre-conditions needed for successful deployment of AI and offer insights regarding the importance of co-creating the future European Health Data Space.

## Introduction

Artificial intelligence (AI) is increasingly affecting our society, holding both hype and hope. AI applications can bring about revolutionary changes in healthcare, governance, research, production, and many other areas. On the one hand, opportunities such as the precision of diagnosis, the prevention of car accidents and more efficient farming techniques are promising. On the other, AI carries several potential risks, such as racial or gender discriminatory biases and infringements of users’ privacy, potentiating stereotypes and often many unknown, unforeseen or unintended consequences that need consideration by society ( Chen *et al.*, 2020; Parikh *et al.*, 2019).

AI has the potential to empower patients and nurses, as most of these tools are aimed at helping individuals adapt their own behaviour and facilitating bidirectional communication between patients and clinicians for more personalised care. It could also enable patients to have a more proactive role in their health management based on a proper functioning electronic health record (EHR), as recommended by the European Commission (EC; C(2019)800) ( European Commission, 2019a). AI tools and systems need to be applied with varying degrees of sophistication to a wide variety of acute and chronic diseases ( Subramanian *et al.*, 2020), such as for diabetes and hypertension, monitoring patients in rehabilitation, ongoing cardiovascular care, mental health care, falls, or dementia and older persons’ care ( Bharucha *et al.*, 2009). These exploitations depend on the validity and reliability of the data stored in EHRs. Furthermore, AI can also be applied in organizational tasks such as scheduling (in hospital and primary care setting), thus ensuring nurses can be relieved of some non-essential administrative tasks to devote more time to direct patient care.

The global healthcare AI market accounted for $0.95 billion in 2017 and is expected to reach $19.25 billion by 2026 ( Statistics MRC, 2019) growing at a compound annual growth rate of 39.7%. The long-term applications of AI in healthcare are still uncertain, as is the exploitation of the EU EHR, mainly due to the lack of trust in new emerging technologies. However, there are very promising prospects for the application of AI in healthcare; coupled with the unprecedented impact on global health of the COVID-19 pandemic, there is a new imperative to rapidly evolve new AI technologies to support health systems under extreme pressures.

The use of AI technologies to deliver care more cost-effectively is an opportunity to relieve the current strained healthcare systems. With a focus on Europe, the European Commission President-Elect Ursula von der Leyen ( European Commission, 2020a) has made clear her ambition to ensure that the next five-year EU legislative cycle harnesses the potential of digital innovation to drive improvements in all aspects of healthcare. To support this, she pledged to create a European Health Data Space ( European Commission, 2019b) and to adopt legislation on AI.

The nursing workforce, which is the largest group of healthcare professionals in the majority of countries worldwide, play a key role in the deployment of the EU EHR and AI technologies in healthcare settings. They also have clear advantage in implementation as they are highly trusted by the public ( GALLUP, 2020). The success of the deployment of the EHR and AI tools in Europe will largely depend on end-users, healthcare professionals and the patients/citizens using them. Engagement and trust will depend on their involvement as co-designers of these technologies with the understanding of the added-value and expertise they bring.

AI is transforming the way we all live, the way we interact with each other, and potentially widens the borders of what is possible and what is not. Within healthcare, the profession that could contribute and benefit the most from AI is the frontline nurses. There are an estimated three million nurses in the EU and six million nurses in the wider European region, which remain desperate for even more highly qualified nurses to join the profession ( Consult QD, 2020).

This article explores the impact of AI on nursing care provision in healthcare, highlighting the importance of co-creation of the EU EHR, and examines the way AI technologies shape the nursing profession today. As such, the art of nursing is becoming a subtle balancing act between the competing demands of human beings, organisation, science and technology. However, the determination to achieve better patient outcomes is a catalyst for nurses in deploying the EU EHR and AI.

## Definition of AI

For the purposes of this article, AI refers to the simulation of human intelligence in man-made machines programmed to be as close as technologically possible to imitate certain human actions. The term may also apply to machines or software programs that are capable of problem-solving and learning. The defining characteristic of all AIs is their ability to take actions towards achieving the best goal they were programmed for; and to continuously learn, deepening its knowledge and accuracy incrementally by the feedback it receives (also known as machine learning).

There are three overarching characteristics shared by all AIs: learning, reasoning, and perception. These three are common characteristics that are attributed to human intelligence. AI can learn from past problem-solving exercises and mistakes (i.e., learning), act and behave in accordance with its surrounding, whether physical or virtual, and the input it receives (i.e., perception); and finally, has the ability to, based on the previous two, come up with the best outcome/solution for its given purpose (i.e., reasoning).

AI, by definition, can be applied to many different uses, sectors, or industries. This links to the cross-disciplinary approach that can be attributed to AI – including mathematics, linguistics, computer science, psychology, and more. This article aims to explore the basics of AI in the context of healthcare, and more concretely, how AI may support the frontline nursing profession to deliver better outcomes for patients and increase the job satisfaction of frontline nurses.

## AI in healthcare

The world of healthcare is constantly developing and increasing in complexity. As the population ages in many developed countries, healthcare systems fall under the pressure of dealing with growing number of patients, a rise in co-morbidities and chronic diseases ( EIT Health *et al*., 2020) as well as medical desertification, which pushes individuals in European regions towards lesser healthy living. However, some scholars argue that the scalable and sustained value of AI for patient care is yet to be realised ( Li *et al.*, 2020). To implement AI in healthcare at scale, the identification of the needs within the complexities of healthcare settings and delivery is essential to steer political and financial commitments. There are four key elements that stand out:

1. The need to have sufficient health data to create a unified, unbiased and consolidated view of the patient’s health status;
2. Creating a compatible aggregation of healthcare provider generated data (e.g. EHR) and patient-generated data (e.g. wearable health trackers);
3. Having an ecosystem in which all stakeholders, including nurses, work together and build on each other’s strengths; and
4. Fostering transparency and integration of newly designed algorithms into existing workflows.

Moreover, in the future, it is expected that the following trends will impact the digitalisation and application of AI tools in healthcare:

1. Telehealth, which is becoming the new standard for care, by fostering digital health consultations and the use of other digital health tools for patients’ self-management of certain conditions. This, however, requires interoperable formats and data, making the EU EHR essential.
2. Hospitals in financial distress, by reducing some costs linked to face-to-face healthcare provision and consultations; and reducing the proportion of severe cases through an earlier detection of pathologies and events.
3. Reduction in regulatory barriers and increase in Medtech-Pharma partnerships.
4. Rise in cybersecurity concerns, as healthcare management and organisation goes digital and online, being more exposed to online crime.
5. Data interoperability is a priority, to foster the cross-sectoral implementation of the EHR and AI tools, as explained by the Healthcare Information and Management Systems Society ( HIMSS, 2019).
6. Increased demand for AI as soon as these new technologies start being successfully applied and producing positive outcomes. Combination of technologies are already seen as a big step forward towards integrating AI in day-to-day health monitoring and health care ( Greco *et al.*, 2020).
7. Remote healthcare, with the aim to provide consultation and monitoring to remotely and potentially instantly to far reached places, reducing delays and quality barriers due to time and distance. For example, from the perspective of COVID 19, new possibilities are being explored ( Fisk *et al.*, 2020). Cloud based tele-robotics and smart homes will also play an important role ( Lanza *et al.*, 2020; Pham *et al.*, 2018).
8. Finally, a renewed focus on mental wellness ( D’Alfonso, 2020; Graham *et al.*, 2019), including being a narrative companion for autobiographical memory ( Dominey *et al.*, 2017), to help in conditions such as depression ( Fulmer *et al.*, 2018), which is seeing wider application globally ( Tran *et al.*, 2019).

The application of AI in healthcare can happen at three levels: 1) at the device level, 2) at the department level, or 3) at the enterprise level. All these link to patient access: AI can help with scheduling backlogs ( Rozario & Rozario, 2020) (hence reducing patients’ waiting times for exams, tests or surgery); reminding of upcoming appointments and no-show planning ( AlMuhaideb *et al.*, 2019); with logistics (distance and weather may impact patients arriving on time, if at all); and scheduling for nurses in community-care settings. On the individual front, the EHR and AI can empower the patient, thus ensuring that chronic conditions are managed daily, thus improving outcomes and reducing costs. This is even more important now that we know those conditions have an aggravating effect on COVID-19 outcomes.

To make EU EHR and AI adoption work, health stakeholders could work towards:

1. Embracing digitalisation in the healthcare sector – a digitalisation degree could be a prerequisite for AI, and digitalisation the benchmark of core processes.
2. Put key enablers in place – fostering leadership sponsorship, implementing sophisticated data strategy, and developing an enterprise portfolio of AI opportunities.
3. Integrate AI seamlessly into existing workflows – involving end-users in the development process.
4. Double down on change management – involving clinical leadership and communicating transparently on the benefits and risks of AI.
5. Awareness and training – design and run initiatives to spread awareness, support adaptation and facilitate the needed skill set. Even a lack of clear role and responsibility when a robotic system is being used, has been seen to be contributing in dissatisfaction and disengagement at the professional front ( Uslu *et al.*, 2019).

## AI in non-hospital settings

While AI tools and applications in healthcare are designed to be used (mostly) at hospital settings (due to their complexity and nature of their operations), they are expanding their operational capabilities to primary care, home and long-term care settings ( Lin *et al.*, 2019). However, logistical issues exist, such as product scalability, inter-system data standardization and integration of the EHR, patient and provider usability and adoption of the EHR, and insurance reform, all of which must be overcome prior to effective implementation of AI ( Aggarwal *et al*., 2020). In non-hospital care, AI tools can be used to strengthen the reliability and efficacy of tele-health (i.e., virtually carried health consultations between patients and healthcare professionals). These electronic interventions can by enhanced if AI tools are used to reduce waiting times and the administrative burden often linked to these.

Moreover, in home and social care settings, AI tools integrated in wearables, mobile and/or home devices may assist patients and citizens monitor their vital signs and manage their own health, for conditions related to heart disease, diabetes, etc. If this is to happen based on a well-functioning EU EHR, AI could help achieve both 1) better patient health outcomes, and 2) alleviate pressure from the healthcare system, by partially delegating some of the responsibility linked to health management (within reasonable terms) to those living with chronic diseases.

## The nursing frontline

Despite the aforementioned potential of AI, in healthcare and more concretely in nursing AI is still in the early phases of design, innovation and deployment. The prospects are promising but they are yet to be realised. AI tools can be integrated into the healthcare workflow to improve effectiveness, efficiency, and utilization. Such AI tools may be vital for quality improvement of services, as well as to scale and diffuse such technologies among teams that may be initially sceptical about their value.

AI has the potential to transform the way nursing care is given. Its effects could be positive and twofold: to be seen by both frontline nurses and patients. AI may allow nurses to better accompany, support and empower patients in their planning and delivery of frontline care. In their daily practice, nurses may benefit from greater access to knowledge and, thanks to AI, constant support for the analysis of complex data. Continuity of information has potential to support the integration of care, alongside its quality and safety.

Moreover, when nurses plan their care, revise medication, and think of nursing interventions, AI has the potential to transform care practices and reduce errors significantly; on the conditions that the health data warehouse, and specifically the EHR, functions to support the workflow of the nurses. It can also ensure constant knowledge sharing/training for every nurse, which is essential when digitalising the healthcare sector.

Considering that the main task of a frontline nurse is caring for their patients, standing by their bedside, being a companion at the end of life, and others – there is an intrinsic human touch that cannot be replaced by anything else – not even the most advanced technology. But in both cases, a robust EHR will augment and supplement nurses’ abilities to perform their duties with the integration of clinical pathways guided by AI.

## AI enabling nurses for direct patient care

Arguably, purpose specific well-designed and implemented EHR and AI have the potential to assist frontline nurses reduce some of their workload in more “automatable” areas (e.g., administrative tasks), assist in focusing attention (e.g. prediction and monitoring of vital signs, Vistisen *et al.*, 2019) and routine tasks (e.g., the hygiene of dependent patients); to in turn give them more time for direct patient care time.

Nurses are integral to the design, development, and deployment of health information technologies. As nurses are always seeking to do a better job serving patient needs, there is a strong argument that EHR and AI are critical in this ongoing digitalisation process. AI can support improvements in care outcomes, patient and healthcare practitioners’ experiences and access to healthcare services. Interestingly, it may also address the burden of administrative tasks, which absorb significant time away from nurses.

In addition, AI technologies can support the shift from hospital-based to home-based care. Hence, ensuring continued integration with community and society. This will require AI to be embedded more extensively implemented in clinical workflows, through the intensive engagement of professional bodies and providers.

## Advanced nursing roles and informatics

Healthcare providers need to assess what advanced nurse practitioners’ distinctive role or contribution can be in introducing or scaling digitalisation in healthcare. They need to take stock of their capabilities, level of digitisation, availability and quality of health data, resources and skills and then define their level of ambition for AI as it fits with their strategic goals ( Adnan *et al.*, 2020). They should also define the enablers they need to put the EU EHR and AI in place. These could include creating an EU health data ecosystem through partnerships to co-develop the right solutions for their community; co-developing a compelling narrative on EHR and AI with patients and practitioners; defining and developing the right use cases jointly with end-users; defining and addressing skill gaps in digital literacy; refining their value proposition for AI talent; addressing the data-quality, access, governance and interoperability issues of the EHR; and shaping a culture of entrepreneurship.

Nursing experience, knowledge, and skills will transition to learning new ways of thinking about and processing information—the nurse will become the co-creator, feedback provider, information integrator, health coach, and deliverer of human caring, supported by AI technologies, not replaced by them.

Moreover, advanced practice nurses may be involved in the following AI-related tasks:

- They can facilitate patients’ understanding of the complex information gathering through the EU EHR and the AI suggestions, that will need to be signed off;
- They may foster the use of clinical data for communication and case management, based on valid and reliable health data in the EHR;
- They may develop data-based guidance that will improve the quality, safety and usability of the EHR data collected and donated for research;
- They can be involved in the upskilling of frontline staff and designing lifelong-learning programmes through continuing professional development, alongside new Master’s and PhD programs with specific focus on digitalisation of the healthcare sector;
- They can play a key role in designing and developing the right EHR and AI interface for nurses and patients.

The EHR and AI has the potential to greatly improve the way nursing care is given and to enhance patients’ experiences. As explained above, AI may assist finding errors in diagnosis and better understand health data captured in the EU EHR.

With nursing input, EHRs and AI may also facilitate:

- Reaching more personalized care, including supporting transcultural nursing through AI and robotics technologies ( Bruno *et al.*, 2017);
- Handling large amount of data and extracting useful patterns;
- Achieving higher accuracy in prediction and monitoring;
- Interfacing with various sensors and tools for sensing and modelling;
- Better training of nurses for different situations, different kinds of conditions and diverse patients’ profiles.

Finally, EHRs and AI can strengthen primary care and help recruit younger nurses, especially if it cuts down on mundane and unpleasant tasks. These are all possible because nurses are natural innovators ready to embrace new technologies that decrease their workload – however, for these to be realised, end-user engagement must be central throughout the whole process of designing the EHR and AI technology.

## The value of co-creation

Co-creation is the process by which end-users (i.e., the people who will be using the new technological advancement frontline) and the technical developers in charge of developing the EU EHR and AI technology (or any other new digital health tool), engage together in a process in which they constantly mutually feed each other and exchange views, needs, expectations and thoughts. The goal of proceeding this way is to ensure that the outcome developed by the technicians is fit-for-purpose and that it addresses needs of the end-users, in ways that are proven useful. This strategy ensures scalability and frontline deployment.

Underlying this, there is research ( Lam & Mattson, 2020) highlighting that by co-creating with the end-users, uncertainties on the new developments may be clarified before the end product is finalised. User-centric design should have the end-user at its heart, meaning that the EHR and AI software should fit seamlessly with the workflow of the frontline end-user and by being used, it will be improved for exploitation.

Moreover, to implement EHR and AI in healthcare multidisciplinary groups of healthcare professionals, including frontline nurses, midwives, physicians, social workers, and occupational therapists should be involved from the beginning of the design process and throughout the implementation of the solution ( Konttila *et al.*, 2019; Li *et al*., 2020; McGrow, 2019; Robert, 2019). Such co-creation is key to the quality of the deliverable, but also to the level of adoption. Too often, new technologies have increased health professionals (and especially nurses) administrative burden and reduced their patient facing time, generating tremendous frustration. Co-creation should ensure solutions bring more patient-facing time, more added-value jobs and ultimately better outcomes. The co-creation framework is also necessary to incorporate different guidelines on inclusive AI, and for AI to respect cultural diversity and pluralism as supported by projects like EU-JP CARESSES ( Bruno *et al.*, 2017).

## The importance of trust and ethics when implementing the EHR and AI

Ethical standards for accessing EHR data for responsible use in AI research and AI health tools are essential. Otherwise, public trust will be undermined. The EC Ethics guidelines for trustworthy AI ( European Commission, 2019c) mentions seven key requirements: 1) human agency and oversight; 2) technical robustness and safety; 3) privacy and data governance; 4) transparency; 5) diversity, non-discrimination and fairness; 6) societal and environmental well-being; and 7) accountability. All of these apply not only to the European context, but to all countries willing to implement digital solutions into their healthcare systems. These seven principles are somehow “universal” as they are needed - the handling of complex health data is a difficult task that requires measures to ensure the full traceability and fair use made of it by nurses (or other end-users).

If all is done correctly, the EU EHR and AI have strong potential to effectively drive changes in the delivery of patient care. For example, one of the uses of AI in clinical analytics is clinical pathway prediction – an essential element for informing nurses’ decision-making processes. Analytics can improve the efficiency and effectiveness of systems that provide and manage nursing care processes and as such continuity of care. The EU EHR needs to boost integrated and continuity of care. The question of AI’s intrinsic risks, such as data protection, information storage, privacy and liability are therefore not a technical one; it is a question of behaviour and individual responsibility in the use of the European Health Data Space.

A key concern of AI practitioners today is managing bias ( Hague, 2019). If computer algorithms are trained using biased information, they will give back biased results. Bias can creep into the EHR and as such into the AI guidance: framing the problem or task to be solved, selecting flawed data to train the system (the data don't reflect current reality or the data represent existing biases), and selecting data that include attributes that will skew the algorithm results. A second aspect of bias to consider is the issue of algorithm transparency. Stakeholders need to look at the AI’s system capability of “explaining” the given results. That is because algorithms manipulate data in a variety of ways, such as sorting, inserting, replacing, or searching for a data attribute.

As AI is growing ever more powerful and entering people’s daily lives, the data collection and donation becomes even more important when designing the EU EHR. Often, transparency in the AI systems is lacking, implying the need to increase the robustness of the EHR data collection and donation. This lack of transparency could fuel practical problems, or even unintended racism, which is why researchers increasingly want to open this ‘black box’ and make AI explainable ( European Commission, 2020b). AI can be more trustworthy to nurses if it is able to manifest itself beyond a black box and if their internal decision making is more traceable, which will not only help in AI assisted collective decision and action but also in better defining the responsibilities, liabilities and authorities ( Gille *et al.*, 2020).

While algorithmic sentencing has already been in use in the United States for a long while now, their adoption in EU countries has been very limited so far – yet AI is already being used in many fields across Europe, with some applications in healthcare. In Europe, the biggest argument against the use of algorithmic sentencing has been the possibility that they may produce some form of bias, like racism, without its designer’s realisation. However, if AI systems and algorithms are made more transparent and “explainable”, these concerns can be tackled.

The EU White Paper on AI ( European Commission, 2020c) calls for explainable AI technologies, with major companies like Google and IBM funding research into it, and the General Data Protection Regulation including the right to explainability for consumers. On top of that, the exact definition of explainability is somewhat unclear, and depends on the situation in which it is applied. An AI researcher who writes an algorithm will need a different kind of explanation compared to a nurse who uses a system for delivering patient care.

While AI stands to be a tool that can help increase efficiency and help nurses make more informed decisions in patient care, there is widespread recognition that a move towards more AI integration in nursing will always be a “machine together with human” formula. Eventually a better human-centred decision will be taken by the human, assisted by AI, based on the robustness of the health data in the EHR. This will ensure that the thoughtful European approach to ethics, health data and patient confidentiality shapes the AI sector, in the same way that data privacy laws share the handling of personal data by companies.

In the field of AI ethics, security standards may need to be updated to accommodate the development of AI. AI standards should help to (1) strike a balance between regulation and innovation, and (2) define robust guidelines for effective competition. At the same time, standards can contribute to policy objectives, e.g. in the area of AI ethics.

Some of the ethical challenges that the implementation of AI systems in healthcare will pose include:

1. Ensuring the privacy and other rights of persons whose data will be used or stored in these systems.
2. Ensuring ethical access to high-quality and inclusive input data sets capable of producing accurate, generalizable, and unbiased results.
3. Ensuring ethical implementation of these tools in all types of healthcare settings – hospital, homecare and long-term care settings.

These issues will need to be addressed through the co-design process between all relevant stakeholders.

## The European White Paper on AI

The EC is so committed to being a driver of AI technologies in Europe that it has published a White Paper on the topic, with the sub-heading of “A European approach to excellence and trust” ( European Commission, 2020c). There are two key elements mentioned there which this article will later explore: trust and excellence. Both of these concepts are intertwined as one is not possible without the other – only if digitalisation, in particular EHR and AI, achieves excellence and delivers to the expectations that end-users’ have put on it, will it be trusted and hence deployed.

Even though this paper refers exclusively at the European dimension, its conclusions and findings are valuable to all the other countries across the globe who are facing the same issues and challenges implementing EHR and AI in their national healthcare systems. The major take-away of this European White Paper is that the right implementation of AI technologies needs an ecosystem of trust among involved stakeholders and end-users. It is difficult to legislate AI, therefore, a framework and rules on AI are important to make it trustful - and “trust” comes from engagement, co-creation and co-design.

In order to accelerate AI development, application and use, and make it an “Ecosystem of Excellence”, it is key to engage the end-user with a coordinated plan on AI, to strengthen multi-disciplinary research and innovation (through networks of excellence); to support testing and experimentation frontline; to promote digitalisation in healthcare sectors; improve skills of the end-users; and promote an ecosystem of trust, linked to a risk-based approach as lack of trust is a main factor holding back a broader EHR and AI uptake.

## Health data spaces

Stakeholders must look at how data spaces are created, regulated and “institutionalised” in countries in which AI tools are being developed and/or implemented. Stakeholders should look at the national and regional regulations of the growing volume of patient’s health data in the EHR. Data can give valuable insights that drive innovation in areas such as nursing, mobility and policymaking. The creation of common data spaces and appropriate data sharing mechanism would allow citizens, businesses and organizations to access non-personalized data from countries, pooled across different key sectors, of which Smart for Health and InteropEHRate are key developments. Data protection and competition laws continue to be applied. Strengthening data quality, governance, security and interoperability is key to build trust among stakeholders, and as such trust in AI.

Moreover, there is a pressing need to engage policymakers and health stakeholders in the discussion on how to make citizens/patients better understand what health data of theirs is being collected and how it is going to be used. In this discussion, we should increase the value of donating data for health research purposes. However, this latter point would require of a greater interest in regulating AI technologies as well as multi-layered data protection approaches. Consequently, the EU EHR multi-layered data protection could drive AI deployment and trust.

The data challenge breaks down into digitising health to generate the data, collecting the data, and setting up the governance around data management. Given the volume of data required for AI, dealing with sensitive data through well-structured data governance is key. On the other hand, AI can also benefit from a combination of data driven and rule-based approaches, in which a better integration of trust can be incorporated and help in making more informed decisions.

Despite all, AI has a strong socio-cultural element attached to it. Europeans are more precautionary and techno-realist than the US or China. It is also cultural by nature, as values do not exist in a vacuum. Systems and values are different, but all health and digital stakeholders need a common ground/agenda, trying to reconcile, to align and combine. Finding the right composition of engagement and forging alliances will be difficult, but we should seek common language, concepts to codesign the EHR and AI systems.

The EU is developing its Health Data Space, joining together 22 member states and four other countries. Its mission is to help member states and the Commission develop an ecosystem where health data is shared more freely, boosting the wellbeing of citizens, the public health system, treatment of illnesses, and the research and innovation activities in Europe. In the future, citizens, communities and companies in Europe will benefit from a protected, secure and seamless access to health data, regardless of where it is stored.

Such an undertaking is much awaited. However, it needs to be bold if we want to tackle long term the current issues of our health systems, rising costs, ageing population and medical desertification. Despite the goodwill of every stakeholder, our current health system is not efficient because it is fragmented and centred on the healthcare provider, not the citizen. The rising costs, the increase in mental disorders, the professional challenges and burn-out confronting more and more health professionals are testimony to those failings.

The shifts that EHR and AI are supposed to deliver should not therefore be applied to specific segments of the current care flows or pathological routes only; it should be used to review the care path in its continuum. On that front, it is critical that the professionals closer to the citizen/patient, the nurses, are equipped to become one of the key drivers of this paradigm shift, and the move from sick care to preventative health. This can reduce prevalence of chronic conditions and increase treatment at home; ensure episodes are identified earlier and reduce their acuteness; and enable monitoring of patients’ post-hospitalisation at home, avoiding re-admissions. Overall, it has potential to improve outcomes and reduce costs.

This implies a shift of resources towards the less glamorous (but effective and efficient) community-care and primary care and the development of a holistic EHR, capturing citizens health and social data to make informed decisions supported by AI. The remaining healthcare resources will be concentrated on the “sick care” approach. This will have a direct impact on costs and outcome and will optimize the use of highly trained professionals towards those cases. It will also free up hospital beds (taken up mostly by chronic condition patients), which will be more easily mobilizable in case of a major event such as another pandemic or a natural disaster.

EU projects, such as Smart4Health and InteropEHRate form a good basis for these EU developments and could empower the co-created European Health Data Space. Smart4Health responds to the European Union Digital Transformation in Health and Care by developing, testing and validating a platform prototype for the Smart4Health citizen-centred health record EU-EHR. The InteropEHRate project also aims to provide European citizens with a complete view of their health history, shareable with health operators and researchers, by means of a multi-alternatives strategy based on 1) the adoption of personal EHRs, 2) the incremental integration of existing EHRs, 3) the support of different levels of interoperability, 4) the usage of blockchain and a decentralized architecture and 5) the human aspects governance.

The idea behind both H2020 projects is to give control of health back to the citizens and patients, by empowering them. By using these health data platforms, citizens will be empowered with electronic health(care) record exchange, personal connected health services, and the ability of data donorship to the scientific community. However, both Smart4Health and InteropEHRate may require an extra push by EU institutions and health stakeholders to ensure their full deployment across the EU’s healthcare systems.

## Integrated EHR - International Classification for Nursing Practice (ICNP) & SOMED CT

AI requires the use of standardised language, while, at the same time, interdisciplinarity imposes the adoption of professional terms for communication. Communication with patients, however, must remain simple and comprehensible. Thus, it is necessary to develop a way to capture and “translate” nursing sensitive indicators into meaningful continuity of care data for the end-users.

A way of approaching this would be by integrating basics of IT computer science (some theoretical frameworks and concepts) into nurses’ curricula (e.g., systems’ theory, information theory, etc.). The use of a standardised professional language (e.g., for semiotics, nursing diagnoses, classification, etc.) is a needed element in the development of nursing care because as each word reflects a concept and the knowledge attached to it.

As still today much of healthcare data are siloed and unstructured, one challenge is a lack of mature data estates to serve as a foundation for AI strategies. Data estates are essential in collecting and storing information in its native format. The need for interoperability between existing EHR is becoming more recognized as an important area of development to leverage Health Data Space and AI platforms.

In this context, there are two elements that could greatly help alleviate the situation, which are the ICNP and SNOMED CT. The ICNP provides a set of terms common to the nursing practice that can be used to record interventions and observations. It provides a framework for sharing nursing data across many settings, including healthcare records. On the other hand, SNOMED CT determines a global of standards for terms in healthcare. It is a computer processable collection of health terms in the form of codes, synonyms and definitions used in clinical documentation and reporting. It can be used in healthcare records and their exchange. As such, the EHR can support nursing research, development and deployment, fostering novel applications and stimulate investment in nursing, creating economic, technological and societal value for EU citizens.

In order to accelerate EHR and AI deployment, scalability and usage, it is important for all stakeholders, IT developers, and frontline nurses’ representatives to strengthen research and innovation through networks of excellence; to strengthen health and social science research; to support testing and experimentation at the frontline; help small and medium-sized enterprises (SMEs) with digital innovation hubs to promote AI in the healthcare sector; improve skills and promote an ecosystem of trust. Within this context, it should be reminded that the nursing profession is the most trusted profession of all (within and outside of the realm of healthcare) in the United States and Europe ( GALLUP, 2020). This level of trust has been achieved by the nursing profession due to their compassion with patients, consistent work, and availability whenever and wherever needed. Within healthcare, nurses are trusted more than the other healthcare professions because nurses are seen as standing alongside their citizens/patients advocating for them ( Krislov, 2020). This position of trust should be taken into account when designing and implementing EHR platforms and AI tools in healthcare – which can be an important ingredient for success. 

In order to ensure that the EU EHR and AI systems are deployed successfully and safely across EU healthcare systems, it is key to:

1. Develop through co-creation robust EU health policies;
2. Support the development of fit-for-purpose electronic health records and improve the interoperability of health data within and between EHR;
3. Equip the healthcare workforce with the necessary skill sets to maximise the positive impact of EHR and AI tools and conduct a comprehensive regulatory assessment of the healthcare professions gearing both the bachelor education and lifelong learning programmes towards digital literacy;
4. Put in place mechanisms to ensure educational assistance to patients to allow them to better understand their health data in the EHR and use AI and empower them to actively participate in the management of their own health status; and
5. Promote open source in order to allow researchers to identify sources of potential bias as well as to validate the results of developed models.

EHR platforms and AI technologies have the potential to complement the art and science of nursing and enhance the safety and quality of healthcare for patients in all settings ( Buchanan *et al.*, 2020). This can only be achieved by the appropriate governance around EHR and AI such as legislative, policy, education, workforce planning, research, development and deployment that is co-designed with nurses as the experts in a broad range of healthcare delivery systems.

## Conclusion

In summary, the EHR platforms and AI systems in healthcare have great potential across many fields and all healthcare professions, improving the outcomes for patients and reducing costs. It brings an opportunity to shift gradually the paradigm of health towards the WHO definition: “Health is a state of complete physical, mental, and social well-being and not merely the absence of disease or infirmity.” Many of the questions that we still have today on its implications relate, precisely, to the degree of maturity of these platforms and technologies impacting the workflow and workload of the end-user, in particular the healthcare provider. However, as these are gradually implemented, always in a process of co-creation and co-design with the respective end-users, doubts on their effectiveness, security and possible biases should be clarified and alleviated as we move forward in co-designing the EU Health Data Space.

Leveraging the advantages and minimising the risks associated with AI is impossible without the presence of a highly trained workforce as well as patients who have the necessary health and digital literacy to engage with and make use of such innovative platforms and tools. Engaging in a whole of society approach using principles of co-design has the potential to maximise the gains of AI.

## Data availability

No data are associated with this article.

## Recommended policy documents for reading

• British Standards. 2016. BS 8611:2016: Robots and robotic devices. Guide to the ethical design and application of robots and robotic

• European Parliament. 2016. Artificial intelligence: Potential benefits and ethical considerations, European Parliament Legal Affairs briefing, Policy Department C: Citizens’ Rights and Constitutional Affairs, PE 571.380 

• Eurostat. 2019. Healthcare personnel statistics - nursing and caring professionals.

• Communication from the Commission to the European Parliament, the European Council, the Council, the European Economic and Social Committee and the Committee of the Regions on Artificial intelligence for Europe (COM(2018) 237 final).

• European Commission. 2018. Communication from the European Commission to the European Parliament, the European Council, the Council, the European Economic and Social Committee and the Committee of the Regions on a Coordinated plan on artificial intelligence (COM(2018) 795 final).

• European Commission. 2018. Communication on enabling the digital transformation of health and care in the Digital Single Market. 

• European Commission. 2019. Digital Europe Programme: a proposed €9.2 billion of funding for 2021–2027.

• European Commission. 2020. Digital education action plan.

• OECD. 2019. Health at a Glance: Europe 2018.

• WHO. 2016. Global strategy on human resources for health: Workforce 2030.
