# Supplementary material for: Leveraging the trust of nurses to advance a digital agenda in Europe: a critical review of health policy literature
Source: Open Res Eur. 2021 May 13;1:26. Originally published 2021 Mar 26. [Version 2] doi: 10.12688/openreseurope.13231.2 (PMC10446062; doi:10.12688/openreseurope.13231.2)
Supplement: Peer review history

# Peer review: Reviewer response for version 2

**DOI:** 10.21956/openreseurope.14733.r26887

**Published:** 2021-05-18

**Authors:** Dorota Kilanska

**Affiliations:** 1 The Department of Coordinated Care, Medical University of Lodz, Lodz, Poland

I approved the changes. Amendments to the article showed the location of its mainstream in scientific research, thus confirming the correctness of the publication goal adopted by the authors.

Is the review written in accessible language?

Yes

Are all factual statements correct and adequately supported by citations?

Yes

Are the conclusions drawn appropriate in the context of the current research literature?

Yes

Is the topic of the review discussed comprehensively in the context of the current literature?

Yes

Reviewer Expertise:

Nursing informatics, coordinated care, PHC, nursing.

I confirm that I have read this submission and believe that I have an appropriate level of expertise to confirm that it is of an acceptable scientific standard.

# Peer review: Reviewer response for version 2

**DOI:** 10.21956/openreseurope.14733.r26885

**Published:** 2021-05-13

**Authors:** Andreas Xyrichis

**Affiliations:** 1 Florence Nightingale Faculty of Nursing, Midwifery and Palliative Care, King's College London, London, UK

Thank you for addressing my and other reviewers' comments. The paper is much improved.

Is the review written in accessible language?

Yes

Are all factual statements correct and adequately supported by citations?

Yes

Are the conclusions drawn appropriate in the context of the current research literature?

Yes

Is the topic of the review discussed comprehensively in the context of the current literature?

Yes

Reviewer Expertise:

nursing research, health policy

I confirm that I have read this submission and believe that I have an appropriate level of expertise to confirm that it is of an acceptable scientific standard.

# Peer review: Reviewer response for version 1

**DOI:** 10.21956/openreseurope.14301.r26671

**Published:** 2021-04-19

**Authors:** Dorota Kilanska

**Affiliations:** 1 The Department of Coordinated Care, Medical University of Lodz, Lodz, Poland

The article presents a broad perspective on the use of data in nursing, their interchangeability through the use of a dictionary that allows describing the patient's status, reporting medical events, but also determining the need for care. AI should be safe and effective for users on this matter the quality of data is very important. The article shows the way of collecting the data used by the huge group of health professionals - nurses, who are main outsourcers of data in health system. Data interoperability is the key to decision support process, and the authors show us this possibility in the last section.

 The publication shows the possibilities of nursing development with the use of IT tools, interoperability and data obtained with the use of international terminology standards for the purpose of improving the quality of care in ensuring comprehensiveness and coordination. The use of terminology standard will support the collection and analysis of data at the UN level, statistics, which can help to compare nurses’ work internationally in terms of improving the quality of care. 

 The article shows the relationship between the use of nursing data in health technology assessment and allows the reader to get acquainted with the possibilities of using AI not only in diagnosis and treatment, but also to support nursing care planning. It will allow us to show how we can link the work of a nurse with the quality of care. 

 In conclusion the article is corresponding to the topic and could be indexed but the only changes I would suggest is that ICNP should be used with ^TM^ abbreviation.

Is the review written in accessible language?

Yes

Are all factual statements correct and adequately supported by citations?

Yes

Are the conclusions drawn appropriate in the context of the current research literature?

Yes

Is the topic of the review discussed comprehensively in the context of the current literature?

Yes

Reviewer Expertise:

Nursing informatics, coordinated care, PHC, nursing.

I confirm that I have read this submission and believe that I have an appropriate level of expertise to confirm that it is of an acceptable scientific standard.

# Response

**Published:** 2021-04-27

**Authors:** Paul De Raeve

**Affiliations:** European Federation of Nurses Associations, Belgium

Dear Dr Kilanska, Thank you for your time and thorough assessment of this manuscript. We have considered your comments in detail and addressed all points in the text. For ease of review, we include below a point-by-point response. Thank you again for your helpful comments, advice and encouragement. We expect this paper to be embraced by the wider community and spark much needed debates and developments in this area. Sincerely, The Authors.

 Reviewer 3: "The article presents a broad perspective on the use of data in nursing, their interchangeability through the use of a dictionary that allows describing the patient's status, reporting medical events, but also determining the need for care. AI should be safe and effective for users on this matter the quality of data is very important. The article shows the way of collecting the data used by the huge group of health professionals - nurses, who are main outsourcers of data in health system. Data interoperability is the key to decision support process, and the authors show us this possibility in the last section."

 - Author response: "Thank you for your assessment, which accurately captures the essence of this paper."

 Reviewer 3: "The publication shows the possibilities of nursing development with the use of IT tools, interoperability and data obtained with the use of international terminology standards for the purpose of improving the quality of care in ensuring comprehensiveness and coordination. The use of terminology standard will support the collection and analysis of data at the UN level, statistics, which can help to compare nurses’ work internationally in terms of improving the quality of care."

 - Author response: "We agree and also expect the paper to be helpful to the wider community, as you have outlined."

 Reviewer 3 "The article shows the relationship between the use of nursing data in health technology assessment and allows the reader to get acquainted with the possibilities of using AI not only in diagnosis and treatment, but also to support nursing care planning. It will allow us to show how we can link the work of a nurse with the quality of care." 

 - Author response: "Indeed, the revised manuscript makes these points clearer."

 Reviewer 3 "In conclusion the article is corresponding to the topic and could be indexed but the only changes I would suggest is that ICNP should be used with TM abbreviation."

 - Author response: "Thank you; the suggestion is headed."

# Peer review: Reviewer response for version 1

**DOI:** 10.21956/openreseurope.14301.r26673

**Published:** 2021-04-13

**Authors:** Andreas Xyrichis

**Affiliations:** 1 Florence Nightingale Faculty of Nursing, Midwifery and Palliative Care, King's College London, London, UK

Thank you for the opportunity to review this interesting discussion essay, examining AI and nursing in the context of EU health policy. Please see below my assessment.

 Abstract: concisely summarises the paper, pointing at the key discussion points of the article. I would remove the mention of (Smart4Health & InteropEHRate) here since this is not explained until later in the manuscript.

 Introduction: provides a sound grounding in the health policy context pertaining to digitalisation and AI, with a link to the nursing workforce. In the last paragraph, the word 'impact' is not quite accurate; perhaps contribution would be more appropriate.

 Definition of AI: explained in simple language; much appreciated. Should AI be distinguished from Machine Learning here? If so, I suggest this is done very briefly so as not to detract from the main focus of the article on AI.

 AI in healthcare (and non-hospital settings): interesting points and literature included here. Is there overlap with the next section (AI in non-hospital settings)? Perhaps both sections could be under the same heading since non-hospital settings are still considered healthcare.

 The nursing frontline: valuable insights shared here. Surprised not to see any supporting literature, though I take it the points made are rather self-evident. Perhaps these papers could be used to support some of your arguments: http://doi.org/10.2196/23933 ^1^ and http://doi.org/10.1097/01.NUMA.0000578988.56622.21 ^2^

 AI enabling nurses: I wonder if this section should be presented under the same heading as the one above (nursing frontline). Both seem to be tackling similar issues, so could be presented together.

 Advanced nursing roles: I enjoyed reading this section. There is much potential in the ideas proposed here.

 The value of co-creation: important points made here, that relevant stakeholders should take into account.

 The importance of trust and ethics: this is key in the article and for me the most important and interesting section.

 The European White paper: this section could be moved further up in the article, perhaps after the 'Definition of AI'.

 Health data spaces: clearly written. The two projects (Smart for Health and InteropEHRate) mentioned in the first paragraph could be linked to the project website.

 Integrated EHR: this is an interesting section, but not sure it forms a strong part of the paper. It puts forth reasonable points, but these have been made before. The section can stay as is, or removed entirely; in either case, I do not think this will have a major impact on the article.

 Conclusion: sound and logical, building on the arguments presented earlier. Overall, this discussion essay makes a nice contribution to current debates on AI and nursing in the context of European health policy; and makes sound propositions to stir further debate, research and policy work.

Is the review written in accessible language?

Yes

Are all factual statements correct and adequately supported by citations?

Yes

Are the conclusions drawn appropriate in the context of the current research literature?

Yes

Is the topic of the review discussed comprehensively in the context of the current literature?

Yes

Reviewer Expertise:

nursing research, health policy

I confirm that I have read this submission and believe that I have an appropriate level of expertise to confirm that it is of an acceptable scientific standard, however I have significant reservations, as outlined above.

## References

[1] : Predicted Influences of Artificial Intelligence on Nursing Education: Scoping Review. *JMIR Nursing* .2021;4(1) : 10.2196/23933 10.2196/23933 PMC832826934345794

[2] : How artificial intelligence is changing nursing. *Nurs Manage* .50(9) : 10.1097/01.NUMA.0000578988.56622.21 30-39 10.1097/01.NUMA.0000578988.56622.21 31425440PMC7597764

# Response

**Published:** 2021-04-27

**Authors:** Paul De Raeve

**Affiliations:** European Federation of Nurses Associations, Belgium

Dear Dr Xyrichis Thank you for your time and thorough assessment of this manuscript. We have considered your comments in detail and addressed all points in the text. For ease of review, we include below a point-by-point response. Thank you again for your helpful comments, advice and encouragement. We expect this paper to be embraced by the wider community and spark much needed debates and developments in this area. Sincerely, The Authors.  

 Reviewer 2:  "Abstract: concisely summarises the paper, pointing at the key discussion points of the article. I would remove the mention of (Smart4Health & InteropEHRate) here since this is not explained until later in the manuscript." 

 - Author response: "Fair point; mention to the two projects removed from the abstract."

 Reviewer 2: "Introduction: provides a sound grounding in the health policy context pertaining to digitalisation and AI, with a link to the nursing workforce. In the last paragraph, the word 'impact' is not quite accurate; perhaps contribution would be more appropriate."

 - Author response: "Thank you, change made."

 Reviewer 2: "Definition of AI: explained in simple language; much appreciated. Should AI be distinguished from Machine Learning here? If so, I suggest this is done very briefly so as not to detract from the main focus of the article on AI."

 - Author response: "Done, in line with other reviewer suggestions."

 Reviewer 2: "AI in healthcare (and non-hospital settings): interesting points and literature included here. Is there overlap with the next section (AI in non-hospital settings)? Perhaps both sections could be under the same heading since non-hospital settings are still considered healthcare."

 - Author response: "Fair enough; two sections now merged under one heading."

 Reviewer 2: "The nursing frontline: valuable insights shared here. Surprised not to see any supporting literature, though I take it the points made are rather self-evident. Perhaps these papers could be used to support some of your arguments: http://doi.org/10.2196/239331 and http://doi.org/10.1097/01.NUMA.0000578988.56622.212"

 - Author response: "Thank you for these articles, which are now cited in the text."

 Reviewer 2: "AI enabling nurses: I wonder if this section should be presented under the same heading as the one above (nursing frontline). Both seem to be tackling similar issues, so could be presented together."

 - Author response: "Fair point; advice headed."

 Reviewer 2: "Advanced nursing roles: I enjoyed reading this section. There is much potential in the ideas proposed here."

 - Author response: "Thank you."

 Reviewer 2: "The value of co-creation: important points made here, that relevant stakeholders should take into account."

 - Author response: "Thank you."

 Reviewer 2: "The importance of trust and ethics: this is key in the article and for me the most important and interesting section."

 - Author response: "Thank you."

 Reviewer 2: "The European White paper: this section could be moved further up in the article, perhaps after the 'Definition of AI'."

 - Author response: "Moved, in line with other reviewer suggestions."

 Reviewer 2: "Health data spaces: clearly written. The two projects (Smart for Health and InteropEHRate) mentioned in the first paragraph could be linked to the project website."

 - Author response: "Done."

 Reviewer 2: "Integrated EHR: this is an interesting section, but not sure it forms a strong part of the paper. It puts forth reasonable points, but these have been made before. The section can stay as is, or removed entirely; in either case, I do not think this will have a major impact on the article."

 - Author response: "Moved following other reviewer suggestions." 

 Reviewer 2 "Conclusion: sound and logical, building on the arguments presented earlier. Overall, this discussion essay makes a nice contribution to current debates on AI and nursing in the context of European health policy; and makes sound propositions to stir further debate, research and policy work."

 - Author response: "Thank you."

# Peer review: Reviewer response for version 1

**DOI:** 10.21956/openreseurope.14301.r26672

**Published:** 2021-04-09

**Authors:** Pamela Hussey

**Affiliations:** 1 School of Nursing Psychotherapy and Community Health, Dublin City University, Dublin, Ireland

Overall I think this paper provides a good summary of the topic and how it relates to the profession, but I think it gets a bit diluted in places and would benefit from some additional signposting. For example listing what the key insights are, and what the key preconditions are which are important to the profession in specific sections might be useful. I have made a few suggestions on edits for restructuring in the body of the material presented and attached to this report. I have also provided some minor editorial suggestions. Finally I think that the definition of AI section needs a little more detail. What is important is distinguishing ML as a type of AI. Overall this is an important paper and I recommend for indexing with some additional editing as per my comments on the attached file.

Is the review written in accessible language?

Yes

Are all factual statements correct and adequately supported by citations?

Yes

Are the conclusions drawn appropriate in the context of the current research literature?

Partly

Is the topic of the review discussed comprehensively in the context of the current literature?

Yes

Reviewer Expertise:

Nursing Informatics, Health Informatics, Standards.

I confirm that I have read this submission and believe that I have an appropriate level of expertise to confirm that it is of an acceptable scientific standard.

# Response

**Published:** 2021-04-27

**Authors:** Paul De Raeve

**Affiliations:** European Federation of Nurses Associations, Belgium

Dear Dr Hussey, Thank you for your time and thorough assessment of this manuscript. We have considered your comments in detail and addressed all points in the text. For ease of review, we include below a point-by-point response. Thank you again for your helpful comments, advice and encouragement. We expect this paper to be embraced by the wider community and spark much needed debates and developments in this area. Sincerely, The Authors.  

 Reviewer 1 Author response

 Overall, I think this paper provides a good summary of the topic and how it relates to the profession Thank you for your positive feedback and encouragement. I think it gets a bit diluted in places and would benefit from some additional signposting. For example, listing what the key insights are, and what the key preconditions are which are important to the profession in specific sections might be useful.

 Thank you for the helpful suggestion. We worked on the signposting in line with comments from other reviewers and took your advice to create a section on ‘pre-conditions’ and expand our Conclusion.

 I have made a few suggestions on edits for restructuring in the body of the material presented and attached to this report. I have also provided some minor editorial suggestions.

 This has been so very helpful -thank you. We have accepted your edits.

 Finally, I think that the definition of AI section needs a little more detail. What is important is distinguishing ML as a type of AI.

 A very good point, also picked up by other reviewers. We have now distinguished between AI and ML more clearly.

 Overall, this is an important paper and I recommend for indexing with some additional editing as per my comments on the attached file.

 Once again, thank you for your time and constructive advice.
